# Advancements in Basal Cell Carcinoma Diagnosis: Non-Invasive Imaging and Multimodal Approach

**DOI:** 10.3390/jcm13010039

**Published:** 2023-12-20

**Authors:** Mircea Negrutiu, Sorina Danescu, Theodor Popa, Monica Focșan, Ștefan Cristian Vesa, Adrian Baican

**Affiliations:** 1 Department of Dermatology, “Iuliu Hatieganu” University of Medicine and Pharmacy, 400012 Cluj-Napoca, Romania; negrutiu.mircea.ionut@elearn.umfcluj.ro (M.N.); abaican@umfcluj.ro (A.B.); 2 Department of Rehabilitation, “Iuliu Hatieganu” University of Medicine and Pharmacy, 400012 Cluj-Napoca, Romania; popa.theo94@gmail.com; 3 Nanobiophotonics and Laser Microspectroscopy Center, Interdisciplinary Research Institute on Bio-Nano-Sciences, Babes-Bolyai University, 400271 Cluj-Napoca, Romania; monica.iosin@ubbcluj.ro; 4 Department of Functional Sciences, Discipline of Pharmacology, Toxicology and Clinical Pharmacology, Faculty of Medicine, “Iuliu Hatieganu” University of Medicine and Pharmacy, 400012 Cluj-Napoca, Romania; stefanvesa@gmail.com

**Keywords:** basal cell carcinoma, dermatoscopy, depth of invasion index, ultrasonography, ex vivo confocal microscopy

## Abstract

(1) Background: The aim of this study was to correlate the diagnostic criteria described in dermatoscopy, ultrasonography (US), ex vivo confocal microscopy, and histology to the most common subtypes of basal cell carcinoma (BCC). (2) Methods: We conducted a prospective study including 46 BCC cases, which were analyzed with dermatoscopy using the Delta 30 dermatoscope and Vidix 4.0 videodermoscope, with US using a high-resolution 20 MHz linear probe, with confocal microscopy, along with histopathological analysis. (3) Results: This study categorized BCC by histological subtype, with nodular being the most common (84.8%) and various other subtypes represented. US measurements of tumor thickness correlated strongly with the histopathological depth of invasion index (DI). Dermatoscopy analysis revealed significant associations between specific features and BCC subtypes. The DI was directly related to arborized vessels but inversely related to short, fine telangiectasias, maple-leaf-like areas, and spoke-wheel areas. The presence of ulceration was directly related to the DI. Confocal microscopy images exhibited several characteristics, including fluorescence, nuclear crowding, peripheral palisading, clefting, increased nuclear–cytoplasmic (N/C) ratio, and a “cauliflower-like” appearance. (4) Conclusion: The advanced detection of BCC through imagistic techniques like dermatoscopy, confocal microscopy, and ultrasound improves the diagnosis and may offer valuable insights for treatment in the future by evaluating lesion characteristics.

## 1. Introduction

Basal cell carcinoma (BCC) is the most common type of skin cancer and originates from the basal layer of the epidermis and adjacent structures. The global incidence of BCC is continuously increasing and varies according to race and geographical factors [1]. Similar incidence rates were observed in Europe, Canada, and Asia, with Australia registering the highest numbers in the world. The biggest increase was observed in Europe, where the incidence increased by 5% annually in the last 10 years [1,2]. BCC appears most frequently after the age of 50 with a predominance for the male sex, but it can also appear at young ages in some syndromes with a genetic predominance, such as xeroderma pigmentosum or basal cell naevus syndrome. The risk factor with the highest incidence is exposure to ultraviolet light, UVA and UVB, thus explaining the predilection for photo-exposed areas [3]. Other risk factors are childhood sunburns, family history of skin cancer, immunosuppression, photosensitizing medication, ionizing radiation, and exposure to arsenic. BCC has a low mortality rate due to its low degree of metastasis, but the morbidity is significant due to invasion and local destruction [4].

Based on the risk of subclinical tumor infiltration and local recurrence, BCC is divided into low- and high-risk tumors, an aspect that guides the therapeutic approach. Thus, for low-risk BCC, one can opt for surgery in the case of nodular BCC, topical therapy with 5% imiquimod or 5% fluorouracil for superficial BCC, while for high-risk BCC (infiltrative, sclerosing) excision surgery with wide margins is required, a model being Mohs surgery, particularly for the head and neck areas [5].

A series of imaging techniques complement the clinical and histological diagnosis. For low-risk forms, especially for those where topical or destructive local therapy is applied, noninvasive imaging diagnosis is accepted. For high-risk forms that are large or present ulcerations, the histopathological examination is mandatory [6]. Dermoscopy is a noninvasive imaging technique that has proven its usefulness in the diagnosis, management, and post-therapeutic evaluation of BCC. Another noninvasive imaging technique is confocal reflectance microscopy (RCM), which can be accessed in centers specialized in skin cancer [6,7]. RCM has proven its usefulness in the differential diagnosis of malignant melanocytic and nonmelanocytic skin tumors, reducing the number of unnecessary invasive procedures. Data from the literature suggest the use of RCM as a complementary method to dermatoscopy, bringing additional details related to the deep layers of the skin [8]. Ex vivo fluorescence confocal microscopy (FCM) serves as a rapid substitute for traditional optical microscopy when inspecting microscopic details of cutaneous surgical specimens. It has been proposed as a fast alternative to Mohs surgery. Unlike Mohs surgery, confocal reflectance microscopy enables the immediate assessment of resection margins postexcision without the need for specimen freezing [9].

Line-field confocal optical coherence tomography (LC-OCT) in skin imaging is finding emerging applications across various skin conditions, particularly in skin cancers [10].

Cutaneous ultrasonography (US) complements the clinical examination, bringing relevant data regarding the size, shape, depth, and vascularization of the tumor [11].

The primary objective of this research endeavor was to establish a meaningful relationship between the diagnostic criteria outlined in various investigative techniques, including dermatoscopy, ultrasonography, ex vivo confocal microscopy, and histology. The focus of this correlation analysis specifically targeted the most prevalent subtypes of BCC, aiming to better understand how these distinct diagnostic methods align in their assessment of these common BCC subtypes. By examining and comparing the criteria used in these different diagnostic modalities, this study sought to enhance our comprehension of how each technique contributes to the accurate diagnosis and characterization of BCC, ultimately providing valuable insights into the clinical assessment and management of this dermatological condition.

## 2. Materials and Methods

We conducted a prospective study during one year (July 2022–July 2023) in patients diagnosed with basal cell carcinoma treated in the dermatology department of our institution. We selected 60 patients, of whom 46 became eligible according to the inclusion criteria (lesion with clinical suspicion of BCC, diameter of the lesion smaller than 3 cm, no previous therapy, tumors that were to be excised) and exclusion criteria (histopathological findings ruling out the diagnosis of BCC, tumors in locations challenging for ultrasonographic analysis (e.g., upper eyelid, ears, scalp), and patients who failed to attend excision appointments for various reasons). The age of patients was between 46 and 87 years (mean 69.9 ± 9.4). The university’s ethics commission and the local hospital approved this study (ID number of the protocol: DEP154/9 May 2022). All study participants were provided with a comprehensive overview of the study protocol and eligibility requirements, and they provided written consent to participate in this study prior to their inclusion. The patients were clinically examined, and the following parameters were assessed: age, sex, tumor clinical diameter, tumor border delimitation, phototype, history of skin tumors, previous burns, and immunosuppression. We considered poor clinical demarcation when clear clinical borders of demarcation between the tumor and healthy skin could not be established (due to perilesional erythema or irregular tumor borders).

Dermatoscopic assessment was conducted using both a Delta 30 dermatoscope and Vidix 4.0 videodermoscope equipped with Vectra software (Version 7.4.7), employing both conventional and polarized light modalities. The images were evaluated by two experienced dermatologists who were unaware of the diagnosis. The main criteria followed were: arborizing vessels, short, fine telangiectasias (vascular), blue-grey ovoid nests, multiple blue-grey globules, maple leaf-like areas, spoke-wheel areas (pigment), ulceration, multiple small erosions, shiny white-red structureless areas, and white streaks (nonvascular/nonpigment-related). The dermatoscopic criteria used in our study were inspired by the work of Lallas et al. [12,13].

High-frequency ultrasound (HFUS) was performed using a high-resolution 20 MHz linear probe (Philips Affiniti 30). The evaluation was performed with the probe perpendicular to the tumor, without applying pressure, using a large amount of gel until it was completely embedded by the same two experienced dermatologists. The US report followed the shape, thickness, level of invasion, composition, border delimitation, presence of hyperechoic spots, posterior enhancement, and tumor vascularization. The ultrasound criteria followed for BCC were those proposed by Bobadilla F et al. [14]. The thickness was measured from the granular layer (below the hyperechoic band of the stratum corneum) to the deepest point of visible tumor infiltration in two axes (longitudinal and transverse). The type of tumor vascularization and the number of vascular pedicles were examined through color Doppler mode. The tumors were excised according to the hospital protocol with 4 mm safety margins. The tissue samples were analyzed at the histopathology laboratory and fixed with hematoxylin-eosin. The histological subtype, depth of invasion index (DI), histological level of tumor invasion, perineural invasion, angiolymphomatous invasion, and excision margins were monitored. We considered the following levels of invasion: I—superficial to basement membrane, II—papillary dermis, III—papillary and reticular junction, IV—reticular junction, and V—subcutaneous fat. Certain excised lesions exhibited multiple histopathological characteristics, such as the coexistence of superficial and nodular features. For the statistical analysis, each characteristic present was taken into consideration. A part of the tumor tissue was fixed in Tissue-Tek O.C.T and frozen at −80 degrees Celsius for 30 min; later, we made sections on ice with the cryotome. We cut sections 5 microns thick and put 4 sections on the blade. For each tumor, 2 slides of 4 sections were made. The slides were analyzed natively under a confocal fluorescence microscope in a specialized center. The fluorescence confocal images of BCC were collected with a super-resolution Re-Scan Confocal Microscopy system (RCM-VIS unit) purchased from Confocal.nl (Amsterdam, The Netherlands) mounted on an ECLIPSE Ti2-E inverted microscope (Nikon) equipped with a Plan-Apochromat 10× objective (N.A. = XX). Specifically, the RCM-Vis images were obtained with a diode laser at 488 nm (TOPTICA Photonics AG, Martinsried/Munich, Germany), captured with a CCD PCO EDGE 4.2 digital camera and, finally, analyzed with the NIS Elements software (version 5.11.02). The FCM criteria of BCC followed were “presence of fluorescence, tumor demarcation, nuclear crowding, peripheral palisading, clefting, increased nuclear-cytoplasmic (N/C) ratio, “Cauliflower-like” appearance”, proposed by Longo C et al. [15].

### Statistical Analysis

Statistical analysis was carried out using the MedCalc^®^ Statistical Software version 22.013 (MedCalc Software Ltd., Ostend, Belgium; https://www.medcalc.org; 2023). Data were presented as mean and standard deviation or frequency and percentage whenever appropriate. Comparison between groups was carried out using the chi-square test or Student t-test whenever appropriate. The Cohen kappa coefficient was used to assess the intermethods reliability. A *p*-value < 0.05 was considered statistically significant.

## 3. Results

This study consisted of 46 cases of patients histopathologically confirmed with BCC, of which 25 (54.3%) were women and 21 (45.7%) were men. Their age ranged between 46 and 87 years, with an average age of 69.9 ± 9.4. The predominant location was on the face (63%), neck and scalp (4.3%), back (26.1%) and anterior chest (2.2%). The dominant phototype was type 3 (67.4%), and 23 (50%) patients had a personal history of BCC. Table 1 contains the patient and tumor characteristics. We classified BCC according to histological subtype (nodular, ulcerated, adenoid, infiltrating, superficial, sclerosing, and others—micronodular, pigmented). Some of the BCCs had mixed subtypes without a clear predominance of one. We included those lesions in both subtypes. Table 2 shows the distribution of the BCC according to the histological subtype.

### 3.1. Ultrasound

We conducted a comparison between the clinical assessment of tumor delimitation and the demarcation provided by ultrasonography. Interestingly, only two cases, constituting 11.8% of those initially categorized as poorly delimited during the clinical examination, were found to be accurately delimited by ultrasonography. The remaining cases exhibited highly consistent results between these two methods, as indicated by a Cohen kappa coefficient of 0.904 (*p* < 0.001), signifying an excellent degree of agreement and overlap.

Furthermore, we observed a strong correlation between the determined tumor thickness (mean value of 2.33 ± 1.32) and the DI measured through histopathology (with a similar mean of 2.3 ± 1.32), supported by a high correlation coefficient (k = 0.996, *p* < 0.001). This indicates that the measurements obtained through ultrasound closely mirrored the actual tumor thickness, as confirmed with histological analysis.

Additionally, our study examined the depth of tumor invasion (papillary dermis, reticular dermis, and hypodermis) as determined by ultrasonography and its alignment with the histological level of tumor invasion. The correlation between these two parameters was notably strong, providing valuable insight into the depth of tumor involvement.

The presence of hyperechoic points and the pattern of vascularity (without/intratumoral/mixt/peripheral) depending on the histological subtype are illustrated in Table 3.

**Figure 1:**
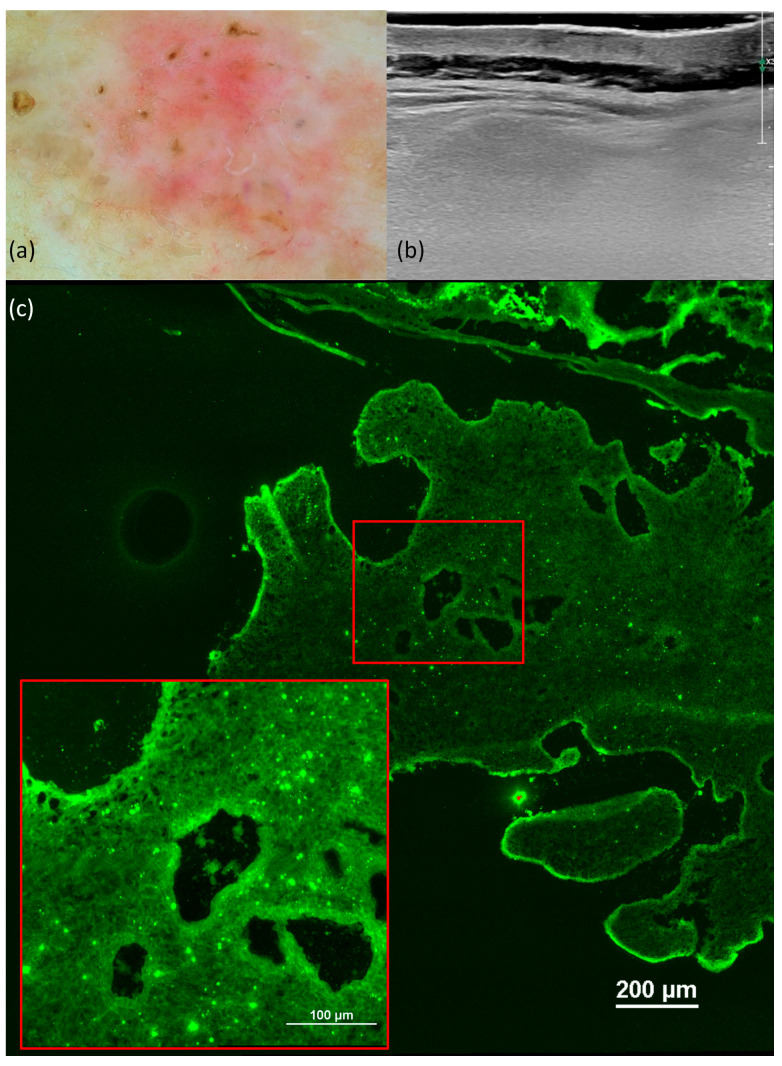
Superficial basal cell carcinoma (BCC). Dermoscopy shows short, fine telangiectasias, maple-leaf-like areas, and white streaks (**a**). Ultrasonography (US) shows a hypoechoic lesion, imprecisely delimited, located at the level of the epidermis and papillary dermis, and without a Doppler signal (**b**). Ex vivo fluorescence confocal microscopy (FCM) shows clefting and peripheral palisading of the superficial tumoral nest (red square) (**c**).

We observed the occurrence of hyperechoic features, a distinctive hallmark of BCC, in all histological subtypes except in the case of superficial BCC (Figure 1). Conversely, when examining sclerosing BCC, we observed the presence of hyperechoic spots in 100% of the cases, although it is important to note that our study had limited data, with only two cases included.

The degree of vascularization exhibited variations among the different histological subtypes of BCC. Specifically, for the nodular subtype, a combination of peripheral and intratumoral vascularization was the prevailing pattern. In contrast, superficial BCC showed an absence of vascularization.

### 3.2. Dermoscopy

**Figure 2:**
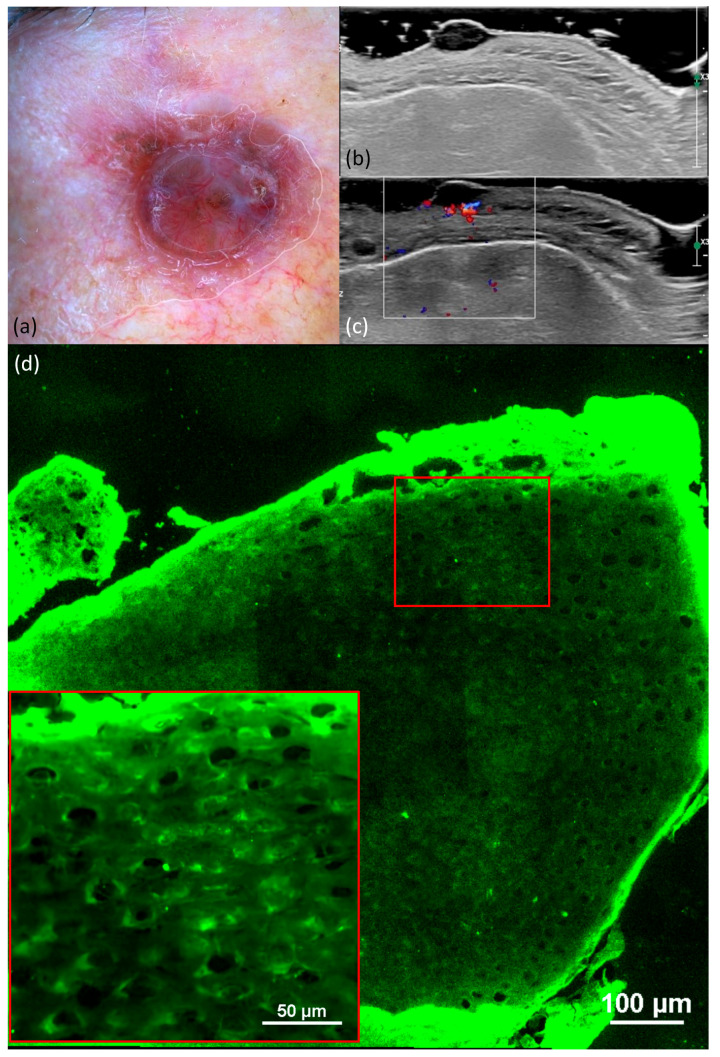
Nodular, adenoid BCC. Dermoscopy shows arborized vessels, blue-grey ovoid nests, and shiny white-red structureless areas (**a**). US shows a hypoechoic, oval, well-defined lesion that reaches the level of the reticular dermis and that contains hyperechoic points (**b**). Doppler mode shows an increase in tumor and peripheral vascularity (**c**). FCM shows well-defined tumor islands, palisading, and clefting (**d**).

We analyzed the main dermatoscopic characteristics according to the histological subtype (illustrated in Table 4). Statistical significance (*p* < 0.05) was established in the correlation between specific characteristics, such as ulcerated BCC and ulceration, adenoid BCC and blue-grey ovoid nests (Figure 2), infiltrative BCC and multiple erosions, superficial BCC and short, fine telangiectasias, as well as maple-leaf-like areas and spoke-wheel areas. Conversely, the remaining characteristics did not exhibit statistical significance.

**Figure 3:**
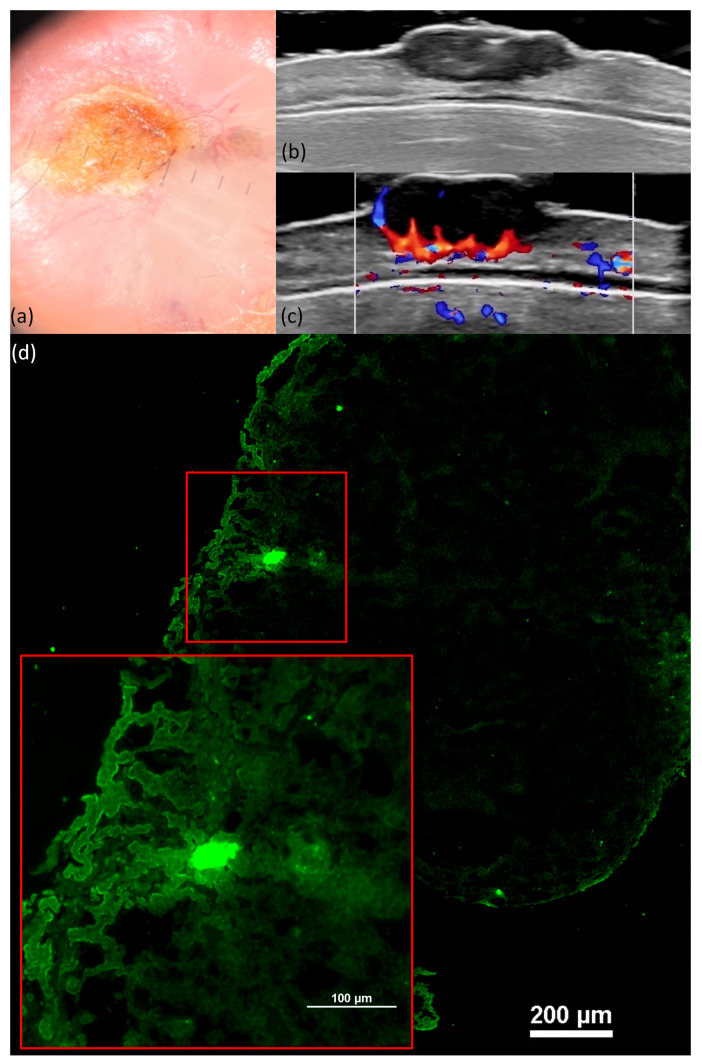
Ulcerated BCC. Dermoscopy shows arborized vessels, ulceration, multiple blue-grey globules, and shiny white-red structureless areas (**a**). US shows a hypoechoic lesion, imprecisely delimited, that reaches the level of the reticular dermis and contains hyperechoic points (**b**). Doppler mode shows an increase in tumor and peripheral vascularity (**c**). FCM shows well-defined tumor islands and a “cauliflower-like” appearance (**d**).

The DI showed a direct proportional variation in size thickness with the presence of dermatoscopic arborized vessels (*p* = 0.001) and an inverse proportional relationship with the presence of dermatoscopic short, fine telangiectasias (*p* = 0.027). Additionally, the presence of maple-leaf-like and spoke-wheel features exhibited an inversely proportional relationship with the DI (*p* = 0.038, *p* = 0.003), while the presence of ulceration (Figure 3) displayed a direct proportional variation with the DI (*p* = 0.005). All of these characteristics are illustrated in Table 5.

The percentage distribution of dermatoscopic aspects according to the histological level of tumor invasion is presented in Table 6.

We observed that certain dermatoscopic features such as arborized vessels, blue-grey ovoid nests, multiple blue-grey globules, ulceration, multiple small erosions, shiny white-red structureless areas, and white streaks were more commonly identified in cases of BCC with a histological level of tumor invasion of IV (invasion into the reticular dermis)–V (invasion into subcutaneous fat) compared to those with a level of II (invasion of the papillary dermis)–III (invasion to the papillary/reticular dermal interface). However, it is important to note that our statistical analysis did not reveal a significant difference between these two groups in terms of the frequency of these features.

### 3.3. Ex Vivo Fluorescence Confocal Microscopy

On the confocal microscopy images, we observed the following aspects: the presence of fluorescence (100%), nuclear crowding (80.4%), peripheral palisading (87%), clefting (67.4%), increased N/C ratio (100%), and “cauliflower-like” appearance (67.4%). The main monitored aspects distributed according to the histological subtype can be found in Table 7.

**Figure 4:**
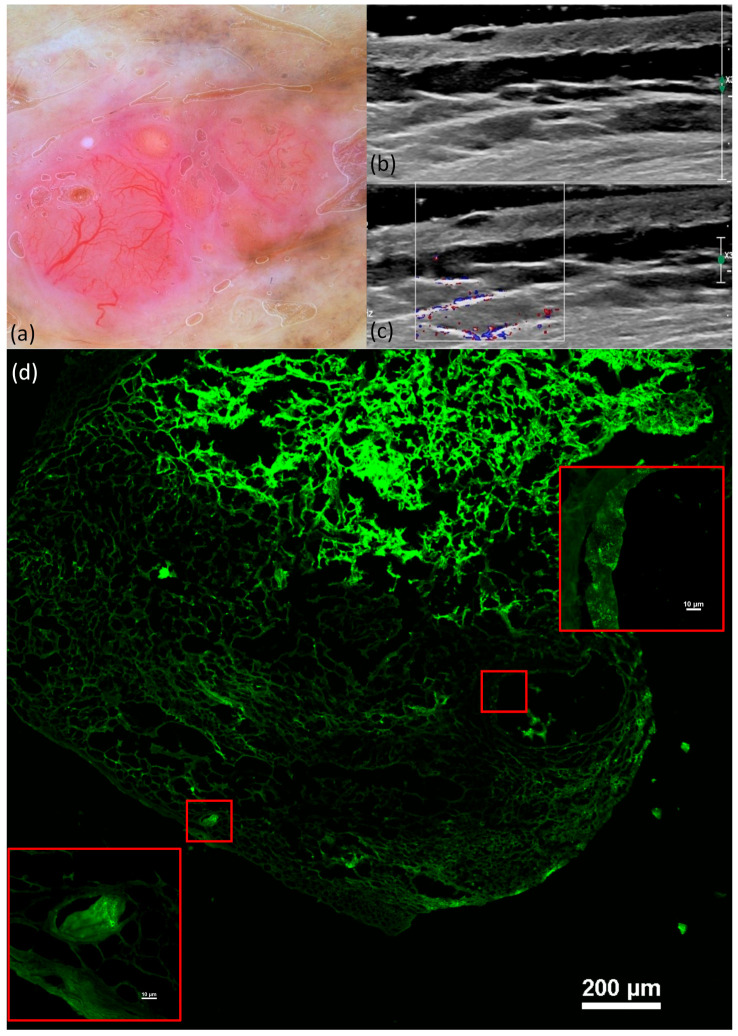
Nodular BCC. Dermoscopy shows arborized vessels and shiny white-red structureless areas (**a**). US shows a hypoechoic lesion, imprecisely delimited, that reaches the level of the reticular dermis (**b**) without a Doppler signal (**c**). FCM shows well-defined tumor islands and a palisading, clefting, and “cauliflower-like” appearance (**d**).

These characteristics were evident in all histological variants of basal cell carcinoma, albeit in differing proportions, with the nodular subtype (Figure 4) exhibiting the highest frequencies. Notably, in cases of sclerosing basal cell carcinoma, tumor demarcation was consistently absent.

**Figure 5:**
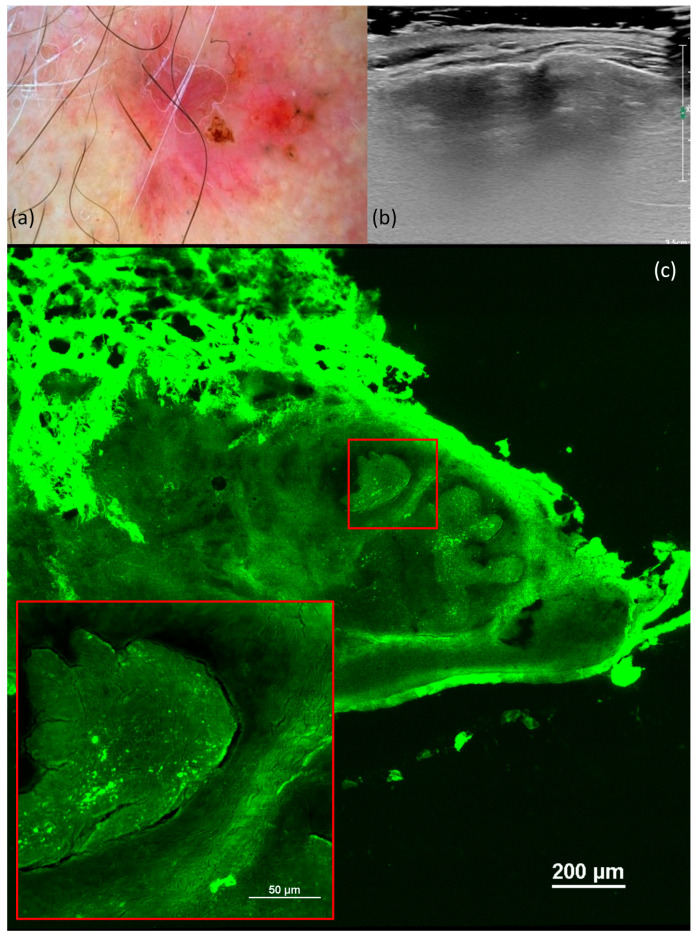
Micronodular, infiltrating BCC. Dermoscopy shows arborized vessels, ulceration, multiple blue-grey globules, and shiny white-red structureless areas (**a**). US shows a hypoechoic lesion, imprecisely delimited, that reaches the level of the reticular dermis and contains hyperechoic points (**b**). FCM shows small fluorescent tumoral nests with the presence of dense stroma and clefting. 4 (**c**).

The DI varied directly proportional to nuclear crowding, peripheral palisading, clefting (Figure 5), and “cauliflower-like” appearance and inversely proportional to the tumor delimitation but without statistical significance (illustrated in Table 8).

The percentage distribution of FCM aspects according to the histological level of tumor invasion is presented in Table 9.

We observed that the features detected in confocal microscopy were more prevalent in basal cell carcinomas categorized as histological level of tumor invasion IV–V compared to those classified as level II–III. However, no statistically significant difference was observed. In terms of fluorescence presence and increased N/C ratio, there was no measurable statistical significance.

The figures in the tables do not add up to 100%, as one lesion can exhibit traits from multiple subtypes. This was the method by which BCC with mixed subtypes could be measured. A BCC can possess traits from various subtypes, and we considered this, incorporating statistical analysis. For example, if a tumor exhibited two subtypes, such as nodular and superficial, the characteristics found would be present in both nodular and superficial BCC. We analyzed the lesion using dermoscopy, US, and FCM without prior knowledge of the histopathological result. It was only later that we correlated these findings.

## 4. Discussion

Dermoscopy, a valuable tool in diagnosing basal cell carcinomas (BCCs), exhibits enhanced sensitivity and specificity when dealing with pigmented BCCs compared to their nonpigmented counterparts. However, diagnosing BCCs located in anatomically challenging regions like the eyes, nose, lips, and ears, or those with poorly defined nonpigmented margins often associated with the sclerosing subtype or recurrent tumors, presents diagnostic challenges. These challenges stem from the inability to precisely assess tumor boundaries, necessitating the exploration of alternative noninvasive imaging methods to gain a more accurate understanding of the tumor’s characteristics.

While histopathological examination is the standard for confirming BCC diagnosis, conventional skin biopsies offer insights only into the specific biopsy site. In cases where a BCC exhibits multiple subtypes, such as infiltrative and superficial components, relying solely on an incisional biopsy from the superficial portion can lead to a potentially inaccurate decision to use a noninvasive treatment method for the entire tumor. This decision may result in incomplete treatment, especially in infiltrative regions. HFUS emerges as a rapid and noninvasive solution with several key functions: selecting the most suitable biopsy sites for maximum diagnostic accuracy, determining the most appropriate treatment modality based on the tumor’s characteristics, monitoring the tumor area after treatment to ensure its response, and any necessary follow-up. US serves as a method to guide us in determining the subtype and depth of the tumor before proceeding with the biopsy [16].

Furthermore, the precise evaluation of surgical margins is of paramount importance, particularly in cases of infiltrative BCCs, which pose challenges in preoperative margin assessment and are associated with higher rates of incomplete surgical excisions. HFUS plays a crucial role in significantly enhancing the preoperative assessment of basal cell carcinoma margins, ensuring more accurate and successful treatments [17].

In the context of this prospective study, we systematically examined basal cell carcinoma using a comprehensive approach that encompassed a range of both invasive and noninvasive diagnostic techniques. Our primary objective was to explore the relationships and associations that exist between these various methods, aiming to gain a more holistic understanding of this type of skin cancer. 

Skin US complements the clinical examination regarding the shape, thickness, extension, vascularization, and consistency of skin tumors. Various subtypes of BCC display unique ultrasonographic features [18]. 

Lesions demonstrating the following characteristics were categorized as having a low risk of recurrence: a regular shape, well-defined boundaries, a consistent internal echo, and confinement within the epidermis and dermis. Conversely, lesions exhibiting the following traits should raise strong suspicions of being high-risk: an irregular shape, indistinct boundaries, and deep infiltration into the subcutaneous tissue. In essence, these indicators pointed to a highly invasive tumor with an elevated risk of recurrence [12].

We found a significant correlation between the tumor thickness measured by US with a linear probe (20 MHz) and the histopathological DI (k = 0.996, *p* < 0.001). Barcaui EO et al., using a 22 MHz probe, obtained an almost similar correlation [19].

Closer correlations were found using lower frequency probes (15–7 MHz) [14] but also using higher frequency probes (30 MHz, 75 MHz) [20], something that proves the usefulness of skin US in choosing the therapeutic attitude as well as the size of the resection margins in the case of a surgical approach. The presence of hyperechoic points as a pathognomonic sign for BCC has been studied considerably in the literature [21]. These points were histopathologically confirmed as calcifications, keratinization, or clusters of apoptotic bodies [22]. In our study, we encountered this aspect in all histological subtypes except in the case of superficial BCC. One reason could be the use of a probe of only 20 MHz, which does not allow for such a good resolution of the surface structures. Instead, in sclerosing BCC, which is a high-risk subtype, the presence of hyperechoic spots was 100%, but the data were insufficient; only two cases were included in our study. Vascularization varied between the histological subtypes of BCC. Thus, for the nodular subtype, mixed vascularization (peripheral and intratumoral) predominated, while for the superficial one, it was absent, so the appearance of vascularization within a previously nonvascularized tumor could mean a negative prognostic factor.

Along with US, dermatoscopy is an imaging technique that helps the dermatological clinical examination. The presence of arborized vessels in BCC is a dermatoscopic criterion with high sensitivity and specificity (over 90%) [23], an aspect found in all subtypes of BCC with different frequencies. In our study, we made a distinction between arborized vessels and short, fine telangiectasias. Arborized vessels were frequently detected in all subtypes of BCC, but short, fine telangiectasias were specific for superficial BCC (*p* < 0.05); a similar aspect was also observed by Chen W et al. [24]. The maple-leaf-like areas and spoke-wheel areas pigmented patterns were most frequently found to be associated with superficial BCC (*p* < 0.01). These two associations were also observed by Tabanlıoğlu Onan et al. [25]. The presence of blue-grey ovoid nests was observed more frequently in larger carcinomas, such as the nodular and adenoid subtypes, and not at all in superficial carcinomas. These represented intratumoral melanin deposits and were directly proportional to the tumor size [26]. Lallas A et al. observed that maple-leaf-like areas, telangiectasia, multiple erosions, and shiny white-red structureless were specific for superficial BCC, while the presence of arborized vessels, blue-grey ovoid nests, and ulceration were specific for nonsuperficial BCC [12], something we noticed as well. The above results were also supported by the variation in the size of the DI. It varied directly proportional to the presence of dermoscopic arborized vessels (*p* = 0.001) and inversely proportional to the presence of short, fine telangiectasias (*p* = 0.027), the presence of maple-leaf-like areas, and spoke-wheel areas (*p* = 0.038, *p* = 0.003). The presence of ulceration was a negative prognostic factor varying directly proportional to the DI (*p* = 0.005), also observed by Verduzco-Martínez et al. [27].

In other words, while these specific dermatoscopic characteristics appeared to be more prevalent in the higher histological level of tumor invasion BCC cases, our data did not show a statistically significant difference between the two groups. 

Confocal microscopy is a relatively new diagnostic imaging technique and can be used in vivo [28] or ex vivo. In our study, we used ex vivo fluorescence confocal microscopy.

Fluorescence confocal microscopy captures fluorescence signals emitted by nucleated cells, including BCC nests and strands, the basal layers of the epidermis, and adnexal structures such as hair follicles (HFs) and eccrine sweat glands. Fluorescence in the dermal region is relatively faint, with observable nucleated cells primarily limited to the tumoral stroma, where they correspond to inflammatory lymphocytes and activated fibroblasts. When viewed using FCM, tumoral cells, HFs, eccrine sweat glands, and the responsive cells within the tumoral stroma exhibit a heightened fluorescence [29].

In contrast to in vivo reflectance confocal microscopy, when examining a sample from its lateral sides and bottom using FCM, it becomes possible to observe muscle and adipose tissue. It is important to highlight that classical histological procedures often alter fat tissue through fixation, while fat tissue remains in its natural state when examined using FCM [30].

According to previous studies, FCM can be useful in predicting the histological subtype of BCC [31].

The main aspects observed with the highest frequency for BCC were the presence of fluorescence (100%), nuclear crowding (80.4%), peripheral palisading (87%), and increased N/C ratio (100%). The DI varied directly proportional to nuclear crowding, peripheral palisading, clefting, and “cauliflower-like” appearance and inversely proportional to tumor demarcation (without statistical significance).

Our observations revealed that the characteristics identified in confocal microscopy were more frequently present in basal cell carcinomas classified as histological level of tumor invasion IV–V compared to those categorized as level II–III. Nevertheless, it is essential to note that these differences did not reach statistical significance.

The typical image of BCC was a tumor formation showing islands of fluorescence with clefting, peripheral palisading, and an increase in the N/C ratio; similar aspects were also observed by Longo C et al. [15]. We encountered different morphological aspects for the subtypes of BCC. Thus, nodular BCC presented nodules with peripheral palisading and clefting. The micronodular subtype presents well-defined round or oval islands and superficial atypical cell proliferation with clefting that forms an axis parallel to the epidermis, aspects also highlighted by Longo C et al. [32]. Unlike other techniques, in our study, we did not use fluorescence fixation [33], and the pieces were frozen in Tissue-Tek O.C.T and later sectioned with a cryotome. A series of studies have shown the importance and usefulness of FCM in Mohs surgery, especially because of its efficiency [33,34,35]. Using our fixation technique, a minimum of 30 min is required, an acceptable time for the diagnosis of the tumor formation as well as for the analysis of the resection margins. In our study, we used a fluorescence confocal microscope used especially in research, but the results are promising and could be extrapolated in future studies and other dermatological pathologies.

We observed a correlation between the histopathological DI and the tumor thickness measured by US and also with the presence of ulceration and arborized vessels (dermoscopy) and with the nuclear crowding, peripheral palisading, clefting, and cauliflower-like appearance (FCM). The clinical relevance of DI is based on the fact that if the tumor is deeper, it will be more difficult to treat topically, even if the European guideline makes a distinction between high-risk and low-risk BCC, which refers to other parameters [6].

The initial data suggest a promising role of LC-OCT in the diagnostic setting for both melanocytic and nonmelanocytic skin lesions, as well as inflammatory and infectious diseases, vascular skin lesions [10], and its ability to detect rare subtypes of BCCs [36]; in addition, LC-OCT showed greater performance when compared to RCM for BCC [37].

There is a scarcity of studies that analyze the same set of patients from all imaging perspectives. A noteworthy aspect is the inclusion of tumors with mixed subtypes, a category often excluded despite its significant representation in clinical practice. The majority of the studies encompassed tumors with a pure component because it is easier to analyze from a statistical perspective.

Our study encountered several significant limitations that are worth discussing in detail. Firstly, one of the prominent constraints was the small number of patients who met the predefined inclusion criteria. The reason for the limited number of eligible participants might be due to the scarcity of dermatologists at our hospital with the necessary expertise in HFUS and FCM procedures. As a result, not all patients with clinically suspected tumors were able to undergo these specialized examinations, leading to a smaller study population.

Additionally, the scope of our study was constrained by the absence of all the subtypes of BCC. The examined sample lacks proper distribution, and the results lack statistical significance, with a notable overrepresentation of nodular BCCs, while other histological subtypes are not adequately represented. This limited representation of BCC subtypes may affect the generalizability of our findings to the entire spectrum of BCC, as different subtypes may exhibit distinct characteristics and behaviors.

Lastly, our study did not incorporate an evaluation of the reproducibility of the assessments, which would have allowed us to examine interobserver variability. Assessing interobserver variability would have provided insights into the consistency and agreement among different evaluators when interpreting the results. The absence of this assessment is a significant limitation as it leaves a gap in our understanding of the reliability and robustness of our findings in the context of different observers or experts.

## 5. Conclusions

BCC has become increasingly prevalent in recent years, emphasizing the need for a swift and accurate diagnosis to facilitate effective treatment. While histopathological examination remains the gold standard, noninvasive imaging techniques like dermatoscopy, confocal microscopy, and HFUS play crucial roles in enhancing diagnostic accuracy. This study stands out for its holistic approach, employing multiple noninvasive methods simultaneously to create a comprehensive diagnostic process for BCC. The goal is twofold: to avoid invasive therapies for superficial cases and to prevent ineffective treatments for invasive forms. Given the challenges of diagnosing BCC, especially on the face, relying solely on clinical and dermoscopic assessments is insufficient. This study also suggests ex vivo confocal microscopy as an efficient alternative to the resource-intensive histopathologic examination for assessing excised tumor margins, promising improved surgical efficiency and accuracy for the benefit of patients and healthcare providers.

## Figures and Tables

**Table 1:** Patient and tumor characteristics.

Variable		Characteristics
Age		69.9 ± 9.4
Clinical diameter		1 ± 0.48
Location	face	29 (63%)
	neck	2 (4.3%)
	scalp	2 (4.3%)
	Posterior thorax	12 (26.1%)
	Anterior thorax	1 (2.2%)
Sex	female	25 (54.3%)
	male	21 (45.7%)
Border delimitation	well	15 (32.6%)
	poorly	31 (67.4%)
Phototype	2	6 (13%)
	3	31 (67.4%)
	4	9 (19.6%)
Previous BCC		23 (50%)

**Table 2:** Distribution of the BCC according to the histological subtype.

Subtype	Number (Percentage)
Nodular	39 (84.8%)
Ulcerated	11 (23.9%)
Adenoid	10 (21.7%)
Infiltrating	4 (8.7%)
Superficial	5 (10.9%)
Sclerosing	2 (4.3%)
Other	6 (13%)

**Table 3:** The presence of hyperechoic points and the pattern of vascularity depend on the histological subtype.

Variable		Hyperechoic Points	Pattern of Vascularity			
			Without Vascularization	Intratumoral	Mixt	Peripheral
Nodular		31 (86.1%)	5 (71.4%)	1 (100%)	18 (81.8%)	15 (93.8%)
	*p*	0.6	0.5			
Ulcerated		8 (22.2%)	1 (14.3%)	0	7 (31.8%)	3 (18.8%)
	*p*	0.6	0.6			
Adenoid		10 (27.8%)	0	0	6 (27.3%)	4 (25%)
	*p*	0.089	0.4			
Infiltrating		3 (8.3%)	1 (14.3%)	0	3 (13.6%)	0
	*p*	1	0.4			
Superficial		0	4 (57.1%)	0	0	1 (6.3%)
	*p*	0.00	0.00			
Sclerosing		2 (5.6%)	0	0	1 (4.5%)	1 (6.3%)
	*p*	1	0.9			
Other		4 (11.1)	1 (14.3%)	0	4 (18.2%)	1 (6.3%)
	*p*	0.5	0.7			

**Table 4:** Dermatoscopic characteristics according to the histological subtype.

Variable	Nodular		Ulcerated		Adenoid		Infiltrating		Superficial		Sclerosing		Other	
		*p*		*p*		*p*		*p*		*p*		*p*		*p*
Arborized vessels	34 (87.2%)	0.2	9 (23.1%)	1	10 (25.6%)	0.3	3 (7.7%)	0.4	0	0.0	2 (5.1%)	1	4 (10.3%)	0.2
Short, fine telangiectasias	5 (62.5%)	0.08	2 (25.5%)	1	0	0.1	1 (12.5%)	0.5	5 (62.5%)	0.0	1 (12.5%)	0.3	2 (25%)	0.2
Blue-grey ovoid nests	10 (90.9%)	1	3 (27.3%)	1	5 (45.5%)	0.043	0	0.5	0	0.3	0	1	2 (18.2%)	0.6
Multiple blue-grey globules	27 (90%)	0.2	8 (26.7%)	0.7	8 (26.7%)	0.4	2 (6.7%)	0.6	3 (10%)	1	1 (3.3%)	1	4 (13.3%)	1
Maple leaf-like areas	12 (92.3%)	0.6	2 (15.4%)	0.4	1 (7.7%)	0.2	1 (7.7%)	1	4 (30.8%)	0.01	0	1	4 (30.8%)	0.04
Spoke-wheel areas	10 (90.9%)	1	1 (9.1%)	0.2	0	0.08	1 (9.1%)	1	4 (36.4%)	0.009	0	1	3 (27.3%)	0.1
Ulceration	16 (80%)	0.6	9 (45%)	0.05	4 (20%)	1	1 (5%)	0.6	1 (5%)	0.3	1 (5%)	1	1 (5%)	0.2
Multiple small erosions	5 (71.4%)	0.2	1 (14.3%)	1	0	0.3	3 (42.9%)	0.009	0	1	1 (14.3%)	0.2	1 (14.3%)	1
Shiny white-red structureless areas	37 (84.1%)	1	10 (22.7%)	0.4	9 (20.5%)	0.3	4 (9.1%)	1	5 (11.4%)	1	2 (4.5%)	1	6 (13.6%)	1
White streaks	24 (82.8%)	1	7 (24.1%)	1	5 (17.2%)	0.4	4 (13.8%)	0.2	4 (13.8%)	0.6	1 (3.4%)	1	4 (13.8%	1

**Table 5:** Variation of the depth of invasion index (DI) compared to dermatoscopic criteria.

Variable	DI	Standard Deviation	*p*
Arborized vessels	2.56	1.27	0.001
Short, fine telangiectasias	1.37	1.47	0.027
Blue-grey ovoid nests	2.81	1.02	0.143
Multiple blue-grey globules	2.51	1.36	0.137
Maple leaf-like areas	1.66	1.07	0.038
Spoke-wheel areas	1.31	0.70	0.003
Ulceration	2.91	1.51	0.005
Multiple small erosions	2.01	1.42	0.534
Shiny white-red structureless areas	2.20	1.22	0.015
White streaks	2.37	1.27	0.667

**Table 6:** Distribution of dermatoscopic aspects according to the histological level of tumor invasion.

Variable	II–III	IV–V	*p*
Arborized vessels	14 (35.9%)	25 (64.1%)	0.1
Short fine telangiectasias	5 (62.5%)	3 (37.5%)	0.2
Blue-grey ovoid nests	4 (36.4%)	7 (63.6%)	1
Multiple blue-grey globules	11 (36.7%)	19 (63.3%)	0.5
Maple-leaf-like areas	8 (61.5%)	5 (38.5%)	0.1
Spoke-wheel areas	7 (63.6%)	4 (36.4%)	0.1
Ulceration	5 (25%)	15 (75%)	0.072
Multiple small erosions	3 (42.9%)	4 (57.1%)	1
Shiny white-red structureless areas	19 (43.2%)	25 (56.8%)	0.5
White streaks	11 (37.9%)	18 (62.1%)	0.7

**Table 7:** FCM aspects distributed according to the histological subtype.

Variable	-	Presence of Fluorescence	Tumor Demarcation	Nuclear Crowding	Peripheral Palisading	Clefting	Increased N/C Ratio	“Cauliflower-like” Appearance
Nodular		39 (84.8%)	32 (86.5%)	32 (86.5%)	34 (85%)	26 (83.9%)	39 (84.8%)	25 (80.6%)
	*p*		0.6	0.6	1	1		0.3
Ulcerated		11 (23.9%)	10 (27%)	9 (24.3%)	10 (25%)	8 (25.8%)	11 (23.9%)	7 (22.6%)
	*p*		0.4	1	1	1		1
Adenoid		10 (21.7%)	9 (24.3%)	9 (24.3%)	10 (25%)	8 (25.8%)	10 (21.7%)	9 (29%)
	*p*		0.6	0.6	0.3	0.4		0.13
Infiltrating		4 (8.7%)	4 (10.8%)	3 (8.1%)	3 (7.5%)	3 (9.7%)	4 (8.7%)	3 (9.7%)
	*p*		0.5	1	0.4	1		1
Superficial		5 (10.9%)	5 (13.5%)	2 (5.4%)	5 (12.5%)	2 (6.5%)	5 (10.9%)	2 (6.5%)
	*p*		0.5	0.04	1	0.3		0.3
Sclerosing		2 (4.3%)	0	2 (5.4%)	2 (5%)	2 (6.5%)	2 (4.3%)	2 (6.5%)
	*p*		0.03	1	1	1		1
Other		6 (13%)	4 (10.8%)	5 (13.5%)	4 (10%)	4 (12.9%)	6 (13%)	3 (9.7%)
	*p*		0.5	1	0.1	1		0.3

**Table 8:** Variation of the DI compared to FCM criteria.

Variable	DI	Standard Deviation	*p*
Tumor demarcation	2.18	1.27	0.231
Nuclear crowding	2.39	1.26	0.368
Peripheral palisading	2.43	1.36	0.090
Clefting	2.53	1.26	0.093
“Cauliflower-like” appearance	2.35	1.43	0.738

**Table 9:** Distribution of FCM aspects according to the histological level of tumor invasion.

Variable	II–III	IV–V	*p*
Presence of fluorescence	19 (41.3%)	27 (58.7%)	-
Tumor demarcation	15 (40.5%)	22 (59.5%)	1
Nuclear crowding	13 (35.1%)	24 (64.9%)	0.1
Peripheral palisading	17 (42.5%)	23 (57.5%)	1
Clefting	11 (35.5%)	20 (64.5%)	0.3
Increased N/C ratio	19 (41.3%)	27 (58.7%)	-
“Cauliflower-like” appearance	11 (35.5%)	20 (64.5%)	0.3

## Data Availability

Data are contained within the article.
